# Community-based health committee initiatives in India: a descriptive analysis of village health sanitation and nutrition committee model

**DOI:** 10.1186/1753-6561-6-S5-O29

**Published:** 2012-09-28

**Authors:** Heikrujam Nongyai Nongdrenkhomba, Banuru Muralidhara Prasad, Biraj Kanti Shome, Achyut Chandra Baishya

**Affiliations:** 1 Regional Resource Centre for North Eastern States, Ministry of Health and Family Welfare, Government of India, India

## Introduction

Primary health care approach is aimed at community participation in understanding the health needs of the community. To address the community participation in institutionalized health services in India, Village Health Sanitation and Nutrition Committees (VHSNC) werer formed at village level under the National Rural Health Mission (NRHM). About 421,892 VHSNCs have been constituted across the country at the village level. The internal program implementation review mechanism has highlighted the lack of clarity over roles and responsibilities of VHSNCs and has stressed upon a detailed research for better understanding. The study presented in this abstract was conducted during the period March to July 2011 in the three of the eight North Eastern states: Manipur, Meghalaya and Tripura.

## Methods

The strata of three districts were selected from Manipur and Meghalaya, and of two districts from Tripura. Furthermore, listing of all VHSNCs in these districts was made along with their village names. Of this list, 10% of VHSNCs were selected based on systematic random sampling method.

A pretested semi-structured interview schedule was used for data collection. The indicators were relating to registers, fund management, community interaction through meetings, interaction with public health institutions, and their experience of importance of VHSNCs. The key informants for the study were the members of the Panchayati Raj Institutions (local elected bodies) and Accredited Social Health Activists (ASHA: female community health workers) who are presidents and member secretaries of VHSNC respectively. The information provided by interviewee was validated through interacting with other members of the VHSNCs. A VHSNC would have about six to seven members. The data validation also included physical verification of registers/records, meeting minutes and schedules at the village.

## Results

The VHSNC constitution in the study state generally followed the national guideline. However the norms for establishing VHSNCs were revised as per the state needs. In Manipur, the norm was based on number of ASHAs rather than number of revenue villages. This dilution led to more number of VHSNCs and therefore more grant in terms of resources from the center. For the ease of financial management, all VHSNCs had to have a bank account in the name of the VHSNC. Only 60% of VHSNCs in the Chandel district of Manipur had opened bank accounts. The funds from the bank were withdrawn at one point of time and this was observed mainly among most VHSNCs in Manipur and Meghalaya state. Overall, utilization of funds was found to be good in all VHSNCs studied. However, the grants that were allocated were erratic and this hampered the activities of VHSNCs on ground. Community level monthly meeting is one of the core activity of VHSNC and the results showed that about 84% of VHSNCs in Manipur, 36% in Meghalaya and 68% in Tripura had organized these meetings regularly. To overcome the resource constraint, many VHSNCs had generated funds through voluntary donations. In Manipur, About 11.4% of VHSNCs in the district were engaged in fund generation activities, ranging from INR 3,000 to INR 8,000.The funds were utilized for construction of community toilets, furniture, safe drinking water, health awareness campaigns and cleanliness activities in the village. Lack of orientation about the roles and responsibilities have also been highlighted by all VHSNCs members interviewed.

## Discussion

We observed that the states had revised the norms related to constitution of VHSNCs taking into account the geo-politico-social context. As a result the number of VHSNCs varied across the states. Secondly, having a bank account for each of the VHSNCs is an important aspect as it can provide details about the number of VHSNCs and the financial transactions. The major drawback in term of financial management is the lack of capacities of VHSNC members to produce finance utilization certificates for the money received on an annual basis. VHSNC model is solely driven by active leadership role assumed by the people’s representatives at the grassroots level. From this study we also realized that some of the VHSNCs had involved in fundraising activities and other involved in providing safe drinking water. All these activities highlight the people’s representatives’ sensitization towards heath needs of the village. We therefore suggest that grassroots level sensitization as well as capacity-building of the members of the committee is essential in successful functioning of VHSNC model.

## Competing interests

None declared.

## Funding statement

None declared

